# Supplementary material for: Developing Burdens in Caring for a Relative with a Cancer Diagnosis: A Qualitative Study of Lived Experiences of Family Caregivers in Saudi Arabia
Source: Nurs Rep. 2025 Jun 26;15(7):233. doi: 10.3390/nursrep15070233 (PMC12300075; doi:10.3390/nursrep15070233)
Supplement: Supplementary file 1 [file nursrep-15-00233-s001.zip › Tabel S2 Participants quotes..pdf]

Interview transcript\ qualitative data analysis

Main- theme	Clarification	Sub-theme	Clarification	Quotation	Interpretation
Antecedent of caregiver burden	During the interview of the participant, they appeared to have a common characteristics , event, or situation previously that facilitated the development of caregiver burden. These antecedents are (insufficient financial resources, multiple responsibility conflicts, and lack of social activity.)	• Insufficient Financial resources	Insufficient financial resources are either due to lack of job, or sometimes the caregiver will leave the job to be able to take care of his relative, or even increased financial expenses due to caregiving demand. Immune compromised patient, especially during chemotherapy sessions needs special attention starting from the food they are eating, to the environment, all should be ensured for its cleanity and safety. Such need may increase the expenses on family caregiver. Moreover, if the patient and the family member is originally from outside the treating hospital, the transportation and leaving beside the hospital in case of discharge may add other financial strain to the family.	Participant 5 reported "*I have to take unpaid leave to take care of her, I don't regret it if she we ask for anything we will do it from the heart, we just want her to gain her health back.*"<br><br>Participant 6 said "*starting from moving to Riyadh for the treatment and then moving back to Tabuk, making the room of my sick son separated from his siblings because he is immune compromised, and off course buying new furniture and spending a lot of money to make the environment clean and safe for him."*<br><br>*In addition , participant 7 said "It's hard at the beginning specially when my mother was newly diagnosed and she has to travel from Haql\ Tabuk to Riyadh for seeking for the treatment we decided to go with her, some of us were able to travel*	participant 5, reported that after consuming her leave balance in her current job as administrative in a school she decided to go for unpaid leave, to be able to stay in Riyadh.<br><br>Diagnosing a family member with terminal disease, in addition to the need to travel with the patient to a tertiary hospital, could be challenging to the family. it may add financial strain, it may also cause multiple responsibility conflict. moving to a new city and leaving home may also raise problems related to adjustment to new environment, especially if the family caregiver lacking social support in the new moved environment. As participant 6<br><br>leaving temporarily or permanently the job can increase the financially burden, moreover the expenses of travelling, in addition to the need to rent a space near to the hospital sometimes, all these factors to financial

				by plain, and some travelled to Riyadh by land, We don't care about financial spendings as long as my mother will be okay" ,	burden. participant 7 moved with all her family members to Riyadh city, even her married sisters came to support their mother during her treatment journey, she said.
				Moreover participant 8 answered " yes,, we spends now more on toys and electronics as you can see(smiling)	the financial expenses may increase due to the caregiving demand of maintaining a safe and clean isolated environment for these special group of patients due to their immunity compression. Moreover the financial resources may decreases as result of being absent, unpaid leaves or even leaving the job to be able to stay with and care for a relative with cancer diagnosis constantly, as participant 8 agreed
				While participant 9 reported "priorities changed, I had to leave my job as seller to care for my mother"	another stress is Leaving the job, can cause deterioration of financial resources of the family caregiver, especially with increased the demand and expenses of care. As participant 9 said:
				On the other side participant 10 stated "*We don't care about the increased expenses, our financial situation is excellent, as I said , no matter of time or money we will spend, the most important , that my*	on the other hand participant 10 verbalized their financial situation is excellent and they don't get attention to it, as the most important matter they care about it is their father, as he said:

				father will be comfortable"	
		• Multiple responsibility conflicts	The family caregiver could be a wife, husband, mother, father, or daughter and son of someone else before assuming the role of family caregiver.	*Participant4 reported While on tears she reported "we are lost we don't know what should we do for my mother, we left everything behind us , my husband, my children, and I don't care for anything in my life except my mother now"*<br><br>*As well as participant 5 stated "I'm married and I have kids alhamdoleleah, I left my kids in Jizan with their father, to be with my sister"*<br><br>*Moreover participant 9 informed "Priorities changed, my mother is the priority before my kids and myself,"*	The additional role of being a family caregiver alongside with other role and responsibility may cause conflicts, leaving the caregiver in stress of feeling lost, and what should and what shouldn't be done, as participant 4 statement<br><br>Participant 5 due to caregiving role she has to leave her children in Jizan to be able to care for her sister, or as she described her" she is the only mother I know".<br><br>Priority has changed, participant 9 decided to take care of her mother while she is hospitalized for around 2 months, living her children in her parent's house with her single, younger sister, participant 9 said:
		• Lack of social Activity	While conducting the individual interview, it was noted that family caregivers came a cross the social support and activity and its relation to their burden earthier positively or negatively. Social support like phone calls and visitation and	*Participant 2 said "My husband understands and feels what I'm going through, thus whenever he is able to come, he will come and will take us for out on pass for a day in a hotel to change my*	Participant 2 reported the long-time hospitalization affected her negatively, as she was feeling alone because she and her husband used to live in Hafer ALbatin, and the husband still their committing to his job.

			offering help is not counted social support by the family caregiver unless they perceived it as a support. Receiving support not necessarily means the family care giver perceived it. Social support if perceived by family caregiver might lessen the stress and burden of caregiving	*mood by changing environment."*<br><br>*On the other hand participant 2 also said "my husband's family, particularly his sister when she called me, she was asking me: how the leukemia started, what cause it?, at the same time my child was just admitted in pediatric ICU after receiving the 1st dose of chemotherapy!"* then after whipping her tears, she continued.<br><br>"*I didn't feel that she was asking and checking on us, I felt she is only curios and intrusive."*<br><br>While participant 6 answered *"With regards to social support Its important but it didn't help me, at the beginning with the stress of the diagnosis and moving from the city, leaving my home and other kids, and taking loan to rent a suitable flat near the hospital made me mor dispirit, having crying bouts and depressed*<br><br>Participant 7 stated *"We have a very good social support systems, my family all around here they came to Riyadh to*	Participant 2 said as well, the call of the relatives is manly not to support rather than curiosity about the situation as she said:<br><br>according to participant 6, the social support she got from other family members is only asking through the phone about her child condition, they didn't offer any other help when she need it<br><br>social support, is a key component of solid relationships and psychological health. social support involves having family members and friends can turn to them in times of need. Social support builds people up during stress time, and often makes

				support my mother and us "<br><br>*On the other hand participant 10 stated "Me and my 2 brothers are with him constantly, we are not leaving him, for more than 4 months we are leaving everything and staying with our father."*	them strong to carry on the caring for a relative with cancer diagnosis. As participant 7 said<br><br>Participant 10 reported that him and his brothers, watching his father constantly, they don't have any time for other activities like social gathering, as he said:
Attributes to caregiver burden	As observed during the interviews, its features that appears repeatedly and its critical attribute of caregiver burden	I. Self-perception	Self-perception is how caregiver perceive themselves while caring for a relative with cancer diagnosis, or what feelings they have about themselves and their new role as a caregiver.	*Participant 5 expressed herself by saying "my feeling about my experience is alhamdolelah good*<br><br>*While participant 3 and 4 said " we are lost, we don't know what to do"*<br><br>*We are only watching my mother dying"*	Feeling good about caregiving role and being satisfied about overall experience is reflecting good self -perception as what participant 5 claimed<br><br>While participant 3 and 4 had bad self- perception about being the caregiver , as their were not able to help their sick mother
		Multifaceted strain	Due to the role of caregiving and sometimes hospitalization, and over time caring, family caregiver might have multidimension strains, as reported by the participant, It may include and not specific to: problems in relationships due to lack of socialization, neglecting their health, sleep disturbance, and feeling fatigued	*Participant 2 said " I had to leave my other child the twin of my sick child to stay for a long time hospitalized and caring for my sick son, and one time I had a sensation that my other child is sick, I had chest tightness as well, when I called my mother and she checked my child; my child was sick and gasping for breath and my parents rush him to hospital*	Participant 2 had other responsibility to another child, the family caregiver left her other child with her mother behind to take care of her sick child, as he was hospitalized for around 6 months with the sick child. She got more stressed when the other child got sick,

				While participant 7 reported saying *"I have another problem my daughter is admitted now in king Fahad medical city and my husband told me that they are suspecting leukemia, since then I'm more stressed and worried but what can I do except to Allah."*	overtime caring and assuming the role of family caregiver, has a consequence of compensating the other roll. The caregiver is mother-father, daughter-son, or wife -husband before assuming the role of family caregiver. Being a family caregiver to a relative with cancer diagnosis, especially during hospitalization period can lead to multiple responsibility conflict, in addition to add more strain and burden to the family caregiver affecting him negatively. Participant 7 reported:
		. Over time	As reported by the participant longtime of caring has either negative or positive impact on caregiver burden. Some family caregivers reported long time caring affected them negatively as they feel they are alone and isolated, while on the other hand other participant stated the long time caring improved them coping and adjusting.	*Participant in calm manner said "Al hamdolelah, now is to much better in comparison to the first month,*	Long time caring for a patient with cancer is devastating, moreover if the cancer patient is a child and needs full assistance with ADls, as participant 8 stated
		. Unsatisfied treatment	During the participant's interview , we came to now that one of the attributes of caregiver burden is the treatment. Either treatment plan as mentioned by some, or treatment team which includes the main physician	*As participant 3 claims "The doctor refused to start up any treatment for my mother as he said she will not be able to tolerate it. We are just sitting here*	Participant 3 reported of being not satisfied with Dr plan of not starting her mother on treatment and upset for not being involved in treatment decision making and

				watching her dying and we can't do anything"	feeling more despite, as she stated
				Additionally, participant 4 said "The team is not consulting us with the treatment, as you can see our mother is sedated with strong meds she doesn't need it because she wasn't on pain, but still the treating team prescribing it to her, and no other interventions, we can't speck to her and she is not aware of us"	Participant 4 was also unsatisfied from the treating team of her mother, they are not involved in decision making, and don't have updated information about what is going on with her mother, as she said:
				While participant 7 said while pointing to a younger sister "My sister the one you see her she is traumatized, whenever the doctor inters the room of my mother she will start to shack up and gets fearful and leaves the room, because the doctor brought the bad news to us and to our mother by saying you have a very aggressive cancer, putting it this way effected my mother and made her stressed more psychologically."	participant 7 reported, that sometimes the treating doctor can be aggressive in the way of disclosing the information either to the patient or to the family caregiver, specially the first time disclosing the information about the diagnosis, as she reported while pointing to her other sister:
				Moreover, participant 10 reported "my father is vomiting constantly and we don't know how to help him, even the doctor decided for	while Participant 10 reported not being satisfied with the treatment and the plan of the doctor, he said:

				discharge as he is palliative case, but how can I discharge him while no medication is stopping the nausea and vomiting , my father is not comfortable but we can't do anything"	
Consequences	Consequences is the result of or the the caregiving to a relative with cancer on either the patient or the caregiver himself. The consequences includes (decease care provision, decreased quality of life, physical health deterioration, physical health deterioration)	V. Decreased quality of life	As discussed by the participants during the semi-structured interview , long time caring for a relative with cancer diagnosis, along with other constrains may decreases the quality of life of the family caregiver. In addition being hospitalized and as a consequences decrease socializations with others may also contribute to decreasesed QOL	*As participant 1 reported "It was harder to my wife as she was the one admitted with our son, she got physically tired, and not sleeping well"*<br><br>*Moreover, participant 8 added I'm leaving with my husband in Riyadh, our family in south of Saudi but I have my cousin and they used to visit me also but not frequently,*<br><br>*While participant 5 reported" I'm not sleeping well and sometimes I forgot even to take my antihypertensive meds, because my sister is the priority, I give all my time for caring to her."*	the constant caregiving may lead to physical and psychological health deterioration. In addition, hospitalization decreases the level of social activity. additionally the family caregiver during hospitalization is less likely to participate in family gathering. Decreased social activity and other factors affecting the QOL of patient and caregiver,<br><br>Long time caring and watching for a relative with cancer diagnosis affecting the quality of life of family caregiver, psychological health, and physical health. family caregiver because of caregiving role may assist the relative patient in activity of daily livings. Attending the needs of cancer patient may also affect the sleeping pattern of caregiver, either by insomnia related to worries, or decreased sleep quality because of the frequent interruptions,

					putting the relative patient needs in more priority than caregiver needs my affect his health by dismissing his appointments if he is hospitalized as participant 5 said:
		I. Decreased care provision	Decrease care provision to the patient happens when the family caregiver is burden with caregiving role.	*Participant 5 stated "one time I forgot to give her the medication, and I gave it late, and I usually forgets to take my medication"*	Family caregiver may be physically tired and unable to assess the patient fully with ADLs, or may forgot an appointment or administrating medications to the receiver as part of decrease concentration or foucus.
		I. Physical health deterioration	Assuming the role of caregiver to a relative with cancer diagnosis can be physically exusting. The family caregiver usually assist the relative with cancer in performing his activity of daily leavings like, eating, showering, dressing, attending follow up appointment, and medication. The family caregivers may have no time for themselves to have rest periods, or to eat well or even to have their meds and follow up appointment if they are with physical health problem due to the demand and the responsibility of caregiving role to a relative with cancer. This physical health deterioration may increase during hospitalization especially if no other family member is supporting and helping.	participant 1 claimed: *"It was harder to my wife as she was the one admitted with our son, she got physically tired, and not sleeping well"*<br><br>additionally participant 9 said" *Even if I will not be sleeping well, who will not be tired if he is watching sick person admitted in hospital!"*	Physical health deterioration is reported as being tired of caregiving, fatigued, poor sleep quality due to hospitalization and its related frequent procedures and interventions<br><br>Long time hospitalization, moreover, caring for a patient with cancer diagnosis that in some situations needs full assistance could be difficult. In addition to the psychological health deterioration, physical health deterioration may occur as a result of prolonged caregiving. Some participants will report changes in sleeping pattern, some will lose wight due to decreased dietary intake,
		I. Psychological health deterioration	Psychological health deterioration starts from the first time the family caregiver	*Participant 1 said "It was hard at the beginning, we were all*	Disclosing the health information of the patient to his family member for the

			hears the diagnosis of cancer. Cancer diagnosis to a family member or relative can be associated with anxiety, sad mood, and depression mood. Another reson could be the long term of caregiving by the family careprovider, as caring for a relative with cancer diagnosis can be stressful. Long time hospitalization and feeling being isolated and the inability to perform social activity may also conterbute to psychological health deterioration	*psychologically stressed, especially when chemotherapy sessions started."*<br><br>*In addition participant 8 reported " it was harder at the beginnings, but know al hamdolelah" then she said "I used to have depression, and crying all the time, seeing the life is black and not worthy, and my daughter will die after all of this."*<br><br>While participant 2 said"*it is very painful experience"" moreover* she continued *"I'm pretending to be strong especially Infront of my parents, I don't want to stress them more, but the moment I will close the phone, I will cry deeply and show my weakness only to Allah, he is the only one can resolve the situation."*<br><br>While crying, participant 3 said "*as you can see everything changed and my mother's health deteriorated very fast since the diagnosis around one month, our life changed."*<br><br>On the other hand participant 6 claimed *"Made me mor dispirit,*	first time, and bringing up the terminal diagnosis of cancer can bring a lot of emotions that can be overwhelming to the receiver<br><br>While Participant 2 described her experience as "painful ", and it was due to multiple factors including Long time hospitalization and caring for a family member with cancer diagnosis affecting psychological health,<br><br>Fast deterioration tin cancer's patient health can be traumatic, affecting the family members quality of life, and may affect physical and psychological health causing its deterioration.<br><br>Participant 6 reported disappointment of the social support from the family when she needs it, special with financial needs as she described.

				having crying bouts and depressed."<br><br>Participant 7 stated "*Me and my all siblings if we will go out of the room we will cry together, and talk about our situations and struggles, but when we have to go back to my mother room, we have to where another face to make her comfortable, because if we are stressed my mother will get stressed."*<br><br>And participant 9 said *"its not easy to hear the one you love is affected by terminal cancer but we are as Muslims accepting whatever from Allah*	Caring for a loved with terminal disease can be associated with overwhelmed emotions, caregiver might be dispirited, hopeless, anxious, having depressed mood, and sometimes cry, as participant 7 stated:<br><br>While participant 9 described her experience as
Religion believes	When person is stressed, it's expected to affect the person in negative way, seldom cases when stressed well show positive attitude and will deeply accept the stress and acts positively. In Eslam , accepting what ever Allah's plan is part of being faithful to Allah.			While she looks calm and comfortable participant 5 reported *"Al hamdolelah, everything from Allah we thank him for it, we are patience, and we are accepting it and no challenges*<br><br>Moreover participant 8 said *"There is a big hope, in such situation the human become powerless, and the only way to become stronger to be faithful to Allah and to become*	being Muslim and with faith in Allah, can bring some reassurance and helps in coping up with stressors, and accepting and dealing with challenges in realistic calmer way,<br><br>while participant 8 reported that her experience improved and she was able to cope up after she became more religious and closer to Allah

				close to Allah, Allah is generous and powerful "	
