# Supplementary material for: Developing Burdens in Caring for a Relative with a Cancer Diagnosis: A Qualitative Study of Lived Experiences of Family Caregivers in Saudi Arabia
Source: Nurs Rep. 2025 Jun 26;15(7):233. doi: 10.3390/nursrep15070233 (PMC12300075; doi:10.3390/nursrep15070233)
Supplement: Supplementary file 1 [file nursrep-15-00233-s001.zip › Table S1 Sociodemographic Characteristics of the Participants in the Focus Group Discussion.pdf]

Table S1: Sociodemographic Characteristics of Focus Group Participants and Cancer Patient Information

Participant	Age	Gender/Nationality	Marital Status	Education Level	Relation to Patient	Employment Status	Monthly Income	Months of Caring	Cancer Diagnoses
1	42	Male/Saudi	Married	High school	Father	Employed	10,000 SR	5	Lung cancer
2	23	Female/Saudi	Married	High school	Mother	Employed	12,000 SR	6	Breast cancer
3	29	Female/Saudi	Single	High school	Daughter	Unemployed	—	1	cancer
4	31	Female/Saudi	Married	Bachelor	Daughter	Unemployed	—	1	cancer
5	42	Female/Saudi	Married	Intermediate school	Sister	Employed	6,500 SR	5	Colorectal cancer
6	39	Female/Saudi	Married	Bachelor	Mother	Employed	12,000 SR	4	Colorectal cancer
7	36	Female/Saudi	Married	Bachelor	Daughter	Employed	13,000 SR	3	Breast cancer
8	28	Female/Saudi	Married	High school	Mother	Unemployed	—	4	Leukemia
9	41	Female/Saudi	Married	High school	Daughter	Employed	6,000 SR	3	Colorectal cancer
10	43	Male/Saudi	Married	High school	Son	Employed	Not specified*	4	Lung cancer

* Monthly income not specified by participant.
